# Assessing Autistic Traits, Hikikomori Tendencies, Pathological Videogaming, and Eating Disorders in University Students: Are Pathological Videogaming and Eating Disorders Gender-Specific Manifestations of the Autism Spectrum?

**DOI:** 10.3390/brainsci14070720

**Published:** 2024-07-17

**Authors:** Barbara Carpita, Benedetta Nardi, Federico Giovannoni, Francesca Parri, Gianluca Cerofolini, Chiara Bonelli, Gabriele Massimetti, Enza Pellecchia, Stefano Pini, Ivan Mirko Cremone, Liliana Dell’Osso

**Affiliations:** 1 Department of Clinical and Experimental Medicine, University of Pisa, 56126 Pisa, Italy; barbara.carpita@unipi.it (B.C.); f.giovannoni10@gmail.com (F.G.); francyparri@icloud.com (F.P.); gianlucacerofolini@gmail.com (G.C.); chiarabonelli.95@hotmail.it (C.B.); gabriele.massimetti@unipi.it (G.M.); stefano.pini@unipi.it (S.P.); ivan.cremone@gmail.com (I.M.C.); liliana.dellosso@gmail.com (L.D.); 2 Department of Law, University of Pisa, 56126 Pisa, Italy; enza.pellecchia@unipi.it

**Keywords:** university students, autism spectrum disorder, autistic traits, hikikomori, video gaming disorder, eating and feeding disorder

## Abstract

In the previous literature, specific attention has been paid to investigate autism spectrum symptoms and traits in university students. In this framework, we aimed to evaluate the presence and correlates of autistic traits, hikikomori tendencies, altered eating behaviors, and pathological videogaming in a sample of Italian university students enrolled in bachelor’s degree courses. A total of 1192 students were recruited via an online survey and assessed with the Hikikomori Questionnaire-25, the Adult Autism Subthreshold Spectrum Questionnaire, the Eating Attitude test-26, and the Assessment of Internet and Computer Game Addiction. Our results highlighted significant differences in the prevalence of autistic traits, social withdrawal tendencies, altered eating habits, and pathological videogame use in university students based on gender, age, parents’ level of instruction, and field of study. A significant effect of the presence of autistic traits and gender on the scores obtained with the other questionnaires was reported. Our results not only support the role of autistic traits as a vulnerability factor for the development of a set of psychopathological conditions but also suggest that gender could modulate this vulnerability, supporting the hypothesis of gender-specific phenotypes in the autism spectrum.

## 1. Introduction

During the last decades, particular attention has been paid to the evaluation of the autistic dimension in university students. Autism spectrum disorder (ASD) is a neurodevelopmental disorder characterized by a range of behavioral, socialization, and communication issues [1]. Despite the fact that ASD is usually considered to have quite an impact on social, occupational and academic functioning, many young individuals with ASD do not have co-occurring intellectual disability or language speech impairment and are able to attend college. It is interesting to note that 0.7% to 1.9% of college students may fit the description of high-functioning ASD [2]. Nonetheless, the investigation of the presence of high-functioning ASD remains pivotal for the subject overall quality of life. Indeed, even though the severity of ASD may lessen in adulthood, those people frequently still need support and have poor social functioning and reduced quality of life [3,4,5]. Moreover, alongside the studies evaluating full-blown ASD, many authors focused on the assessment of autistic traits in the university populations. Indeed, subthreshold autistic traits appear to be particularly represented in some groups such as students of scientific courses [6,7] and are associated with significant clinical correlations, like greater vulnerability towards the development of psychiatric disorders and suicidal ideation and behaviors [8,9,10]. Historically, compared to the general population, autistic people were less likely to pursue higher education, but things are starting to change in this regard, in part because of funding for diversity and equality initiatives as well as wider participation goals [11]. The percentage of autistic students earning a higher education degree is rising. In the US, autistic students at universities make up between 0.7 and 1.9% of the student body [2], while in the UK, the percentage is slightly higher, rising from 1.8% in 2004 to 2.4% in 2008 [12]. Moreover, recent studies have valuated the presence of autistic traits and camouflaging behaviors in the Italian university population, raising knowledge about the significant incidence of autistic traits in the university population [13,14]. Therefore, it is necessary to take into account the unique needs of autistic pupils in a timely manner because it is anticipated that these numbers will rise even more in the foreseeable future [15]. Indeed, without proper integration and support, evidence suggests that fewer than 40% of autistic students graduate from college, making this an extremely crucial issue [16,17].

University students indeed represent a population of extreme interest for mental health studies. This population is going through a period of life characterized by many challenges, such as changes, the exploration of new contexts, and a transition towards various possible career choices in life, ultimately being a phase in which an individual’s path has yet to be completely determined [18]. Subjects must also adjust to new life issues, such as interactions with new colleagues, moving to another city, living without parental support, and eventually starting to work to support the university costs [18]. On the other hand, university students’ age range is also that of the onset of many mental disorders [19]. A recent study pointed out that, generally, the onset of mental disorders occurs in 75% by the age of 25 [20], and it has been suggested that mental health conditions may be eventually precipitated by the entrance into the complicated world of university itself [21]. Moreover, recent research studies have highlighted how, due to the presence of gender-specific symptoms, such as seemingly better social functioning, ASD in females may frequently go undiagnosed [22,23,24]. Indeed, compared to males, autistic females not only have a higher social drive and integrate better with peers, but they also exhibit alternative types of restricted interests, such as appreciating fiction, spending time with animals, or focusing on food and diet [22,23,24]. In light of this evidence, many authors suggested the existence of a female presentation of ASD that occasionally overlaps with other mental illnesses that are thought to be more prevalent in females, thus explaining the historically known gender gap in ASD diagnosis. In this framework, the first research studies on the female phenotype of ASD focused on its overlap with anorexia nervosa, with which it shares numerous aspects including cognitive rigidity, rigidity in set-shifting tasks, diminished emotional awareness, and difficulties in theory of mind [23,24,25,26,27]. Although anorexia nervosa and ASD are different diagnostic categories, several research studies have now confirmed the many similarities between the two disorders as well as their familiar aggregation, extending it also to other kinds of eating disorders [28,29,30]. Moreover, eating disorders have been widely studied in university students, sometimes hypothesizing a connection with the chosen course of study, with the changes to be faced at the beginning of university life or with the tendency towards perfectionism and the need to satisfy social expectations [31,32,33].

Furthermore, particular attention has recently been paid to two emerging pathologies that primarily affect young adults, video gaming addiction and hikikomori. Videogame addiction is characterized by a deficit in controlling gaming activities, in prioritizing gaming over other daily activities, and continuing and intensifying the gaming despite negative consequences, with an overall impairment of functioning [1]. Recent studies aimed to evaluate the prevalence of the disorder in student populations with heterogeneous results, sometimes even reaching a prevalence of one-sixth [34,35]. Indeed, video gaming disorder and internet gaming disorder have been vastly investigated in university students in various countries, raising awareness and concerns. In particular, a recent study in the Iranian university populations has highlighted an overall prevalence of 4.3% as well as many associated risk factors such as male gender, playing online games, and having access to different gaming devices [36]. Similarly, in Saudi Arabia, the estimated prevalence of gaming disorder in university students was reported to be 21.5% [37]. A recent study on American campus students highlighted how 39.4% of the students played videogames for more than 15 h per week and 5.3% also showed more than five symptoms of dependence. Moreover, students with problematic gaming behaviors reported higher suicidal thoughts and behaviors and greater depressive and social phobic symptoms [38]. Even in Italy, the data show how this is a rambling problem, reporting a risk for developing a gaming addiction in 84.61% and an overall prevalence of the disorder estimated at 14.9% in a 2018 study [39]. The evaluation of excessive videogame use in young adults appears to be particularly relevant in light of its association with lower academic results, self-esteem, and life satisfaction, as well as higher school dropout [40,41]. On the other hand, hikikomori has been defined as a form of pathological withdrawal or social isolation lasting longer than six months, associated with a significant functional impairment or distress [42,43]. Even though hikikomori has for long been thought as a condition bound to the Japanese culture, in recent years, international research has described cases from different countries of the world [42,43,44,45,46,47]. While a sense of hopelessness for finding a place in society has been considered a risk factor for developing hikikomori, the spreading of this condition all around the world was hypothesized to be also linked with the increased difficulties in finding employment and financial security due to the economic stagnation [48]. To date, studies on hikikomori in university students are still in their infancy. However, a growing body of research studies are highlighting how this has become a syndrome of global concern and how the student population is particularly affected by it. In particular, Italian and Japanese studies have described how these behaviors are present in the university population and how they represent risk factors for the development of other mental disorders [49,50], while an international study has described their prevalence in Americans, Singaporeans, and Nigerians [51]. Overall, hikikomori has been described in university students, with up to 22% of this population possibly being at risk of developing hikikomori-like social withdrawal [50], and it seems to be linked to higher dropout rates, lower academic outcomes, worse social achievement, and higher risk for the onset of psychiatric symptoms [52,53,54,55]. Several studies stressed that these two conditions seem to be deeply intertwined: social withdrawal may facilitate the risk of developing videogaming addiction as a form of entertainment but also as a method to maintain social relationships while remaining at home. The pathological use of videogames may enhance the risk of social withdrawal due to the large amount of time spent in the activity [36]. Moreover, both conditions have been linked to the presence of the autism spectrum as a possible predisposing factor [56,57,58,59].

In this framework, the aim of the present work was to evaluate through self-administered questionnaires the presence and correlations of autistic traits, hikikomori traits, altered eating behaviors, and pathological videogaming in a sample of Italian university students enrolled in bachelor’s degree courses. Considering the above-mentioned literature, this specific population was chosen considering that subjects at the beginning of their academic path may show greater vulnerability to develop distress related to psychopathological symptoms and traits. Furthermore, the study also focused on investigating how age, parental education level, and type of academic field may influence the behavioral symptoms assessed. In particular, the decision to take these variables into consideration arose from the scarce but promising literature on the topic. In fact, despite not always concordant results, since the first conceptualization of ASD, scientific studies have investigated and hypothesized a correlation between the parents’ level of education and the presence of autistic traits in their offspring. Indeed, while some authors excluded the relevance of parental education as a risk factor for the presence of higher autistic traits, others highlighted the presence of a parental higher educational degree as a pre-natal risk factor for the development of ASD. Similarly, some authors have also hypothesized a possible connection between autistic traits and scientific aptitude, highlighting a relationship between pursuing a science degree and showing higher autistic features. Finally, our decision to include age related to the course of study was born from the hypothesis that subjects with high autistic traits could be penalized by the university system, encountering greater difficulty in adapting to the new context and resulting in a slowdown in their studies. Lastly, a specific focus was also paid to the possible impact of autistic traits and gender on hikikomori, pathological videogaming, and altered eating behaviors. 

## 2. Materials and Methods

### 2.1. Participants

Students of the University of Pisa received an email encouraging them to participate in the survey.

Subjects were requested to fill out anonymously an online form with self-report psychometric tools and sociodemographic information such as age and gender, after giving their consent to participate. To take part in the study, participants were neither paid nor given any additional benefits. Every student had the option to ask to speak with a psychiatrist in order to discuss the investigated psychopathological dimension in more detail. The local Ethics Committees approved the recruitment and assessment procedures, and the study was conducted in accordance with the Declaration of Helsinki.

### 2.2. Psychometric Instruments

#### 2.2.1. Hikikomori Questionnaire-25 (HQ-25)

The HQ-25 is a self-administered questionnaire used to assess the risk for a form of severe social withdrawal. Originally developed in English and Japanese, the HQ-25 consists of 25 questions evaluated on a scale from 0 to 100, where higher scores indicate a higher risk for hikikomori. In the original study, three main domains were identified in the scale: socialization (exploring a person’s tendency to avoid or withdraw from social interactions), isolation (assessing the degree of isolation and lack of participation in social activities or interpersonal relationships), and emotional support (examining the perception of insufficient emotional support from others). 

Internal consistency, test–retest reliability, and convergent validity were all satisfactory. A cut-off score of 42 (out of 100) was proposed for a risk of hikikomori [60].

#### 2.2.2. Adult Autism Subthreshold Spectrum (AdAS Spectrum)

The AdAS Spectrum is a self-report tool created to assess subjects without intellectual disabilities for the presence of full- and subthreshold autistic symptoms and features [61]. The 160 items in the questionnaire are divided into seven domains: *childhood/adolescence, verbal and nonverbal communication, empathy, inflexibility and adherence to routine, rumination and restrictive interests*, and *hyper-hypo reactivity to sensory input*. According to the authors, the tool has two verified threshold values: a cut-off score of 43 that denotes the existence of clinically significant autistic traits, and a cut-off score of 70 that denotes full-blown ASD symptoms [62].

#### 2.2.3. Eating Attitude Test-26 (EAT-26)

The EAT-26 is a widely used self-report measure designed to assess symptoms and traits related to eating disorders. The questionnaire has been employed in numerous studies as an early screening tool to identify individuals with eating disorders. The EAT-26 is composed of 26 questions, divided into three domains (*dieting, bulimia and food preoccupation, and oral control*), and rated on a scale from 0 to 100, where higher scores indicate more severe symptoms [63,64].

#### 2.2.4. Scale for the Assessment of Internet and Computer Game Addiction (AICA-S)

The AICA-S is a validated tool with robust psychometric properties designed to distinguish between regular, excessive, and pathological videogame use. It is composed of 14 items which investigated several features of the condition, including those related to time spent in gaming, tolerance, craving, loss of control, and withdrawal. Answers are organized in a Likert scale [65].

### 2.3. Statistical Analysis

Every statistical evaluation was performed with Statistical Package for Social Science (SPSS) version 26.0.

For the aim of the present work, students enrolled in three-year bachelor’s degree courses were included in the analyses.

A first *t*-student test was used to compare the AdAS Spectrum, HQ, EAT-26, and AICA-S total and domain scores in the sample based on gender. 

A subsequent *t*-student test was used to compare the same variables in the sample based on the age above or below 23 years. The choice to use this threshold age value was motivated by fact that it is considered to separate subjects who are taking longer than expected to graduate and subjects who are up to date with their studies, for bachelor’s degrees. Specifically, the average age at which a student is expected to graduate from high school is 19 years old. According to the 2023 AlmaLaurea reports, 84.1% of those enrolled in a three-year degree course enrolled at most one year later than the “canonical” age, defined as 19 years. Graduation was expected at the age of 23–25 years; in fact, in the bachelor’s courses, the average age of graduates in 2023 was 24.5 years. However, according to data provided by the Italian Ministry of Education, about 40% of students experience some sort of delay. Lastly, student-workers, i.e., all graduates who have completed work experience during their university studies, make up approximately 58.3%.

The other two *t*-student tests were used to compare the questionnaire scores in the overall sample based on the educational level of the parents; in particular, for this purpose, a threshold value of 13 years of schooling was used to distinguish those with a higher educational level than a high school diploma.

ANOVAs followed by Bonferroni post hoc tests were then used to compare AdAS Spectrum, HQ-25, EAT-26, and AICA-S total and domain scores in the overall sample based on the type of study course. For this purpose, the study courses were divided into three groups: Sciences, Humanities, and Applied and Social Sciences. In particular, the Sciences group was formed by Biology, Chemistry, Physics, Informatics, Mathematics, and Earth Science students; the Humanities group included Civilizations and forms of knowledge and Philology students, and the Applied and Social Sciences group comprised Engineering, Medicine, Economy, Pharmacy, Law, Agricultural Sciences, Political Science, and Veterinary students. In particular, we chose to distinguish students from the so called “pure sciences” from those of the “applied science”. We decided to include in the Science group exclusively those disciplines interested in developing scientific theories to improve the understanding and prediction of natural phenomena and which therefore focus on fundamental (theoretical) studies regardless of practical application. Similarly, we decided to include students from Engineering, Medicine, Economy, Pharmacy, Agricultural Sciences, Science, and Veterinary courses in the group Applied and Social Sciences as they use the scientific method and the knowledge obtained through the conclusions of the method to achieve practical objectives.

We then performed three two-way ANOVAs to study the effect of the presence of clinical symptoms of ASD (based on the AdAS Spectrum threshold score of ≥70) and gender on the HQ-25, EAT-26, and AICA-S total scores. Subsequently, two two-way MANOVAs were performed to evaluate the effect of presence of ASD clinical symptoms and gender on the HQ-25 and EAT-26 domains, respectively. For this purpose, the HQ-25, EAT-26, and AICA-S total scores (one for each two-way ANOVA) and domain scores (for the MANOVAs) were used as dependent variables and gender and the presence of clinical symptoms of ASD as independent variables.

Then, in order to determine which characteristics—gender and the presence of ASD clinical symptoms—best predicted the risk of hikikomori, pathological videogame use, and altered eating behaviors, three decision tree models were run. The method of growing chi-squared automated interaction detection (CHAID) was applied. With this method, we were able to assess the interactions between many elements and create a decision tree model that graphically depicts the outcomes as an inverted tree. All of the cases are contained in the root node of the model.

Next, the main differentiating variables are then found, and at each stage, CHAID selects the independent variable that has the strongest relationship with the dependent variables in order to construct the tree. Next, the tree is built by identifying the key discriminating variables: at each step, CHAID chooses the independent variable that shows the strongest interaction with the dependent variables. When there is no reported difference with respect to the dependent variable, the model also combines the categories described by the predictors. The model additionally combines the categories that the predictors have defined when there is no reported difference concerning the dependent variable.

## 3. Results

The overall sample was made of 1192 university students, attending three-year bachelor’s degree courses. The sample consisted of 577 (48.4%) males and 615 (51.6%) females.

The *t*-student test results reported in Table 1 showed how males scored significantly higher in the AdAS Spectrum empathy domain, AICA-S total score, and HQ-25 isolation and emotional support domain scores compared to females. On the other hand, females reported significantly higher scores in the AdAS Spectrum hyper-hyporeactivity to sensory stimuli domain, in all EAT-26 domains and total score, and in the HQ-25 socialization domain.

Table 2 shows the results of the *t*-student test analysis that compared the AdAS Spectrum, AICA-S, EAT-26, and HQ-25 scores in subjects who were 23 years of age or older and subjects who were younger than 23 years of age. This threshold age was chosen because it is thought to distinguish between people who are keeping up with their studies for a bachelor’s degree and those who are taking longer than expected to graduate. The results showed that subjects who were 23 years old or older reached higher scores in the AdAS Spectrum childhood and adolescence domain, AICA-S total score, and all HQ-25 domains and total score.

Table 3 and Table 4 report the results of the *t*-student test analysis comparing the questionnaires scores based on the level of instruction of the fathers and the mothers of the subjects, respectively. Interestingly, no significant differences on the scores emerged based on the educational level of the fathers. Conversely, subjects whose mother had more than 13 years (13 years is the time required to achieve a high school diploma in the Italian education system) of schooling scored higher on the AdAS Spectrum *verbal communication* and *non-verbal communication* domains and total scores. 

Lastly, the ANOVA results (Table 5) showed that Humanities and Science students scored significantly higher than Applied and Social Sciences students in the AdAS Spectrum questionnaire without significant differences between the two groups with the exception of the *hyper-hyporeactivity to sensory input* domain, for which Humanities students scored significantly higher than Science and Applied and Social Sciences students without a significant difference between the latter, and the *verbal communication* domain, for which Science students scored significantly higher than Applied and Social Sciences, but Humanity students’ scores did not significantly differ from either group. Similarly, Humanities and Science students scored significantly higher than Applied and Social Sciences students in the HQ-25 questionnaire without significant differences between the two groups with the only exception of the *emotional support* domain, for which no statistical difference emerged among the three groups. AICA-S total score was found to be significantly higher in Science students compared to Applied and Social Sciences students, while Humanities students’ scores did not significantly differ from either group. Lastly, no significant differences were reported in the EAT-26 questionnaire with the only exception of the *oral control* domain in which Humanities students scored significantly higher than Applied and Social Sciences students.

**Figure 1:**
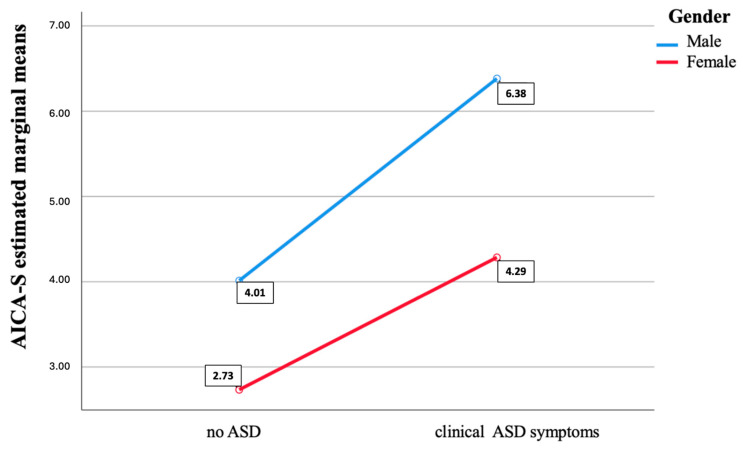
Effect of the presence of clinical ASD symptoms and gender on AICA-S total score.

**Figure 2:**
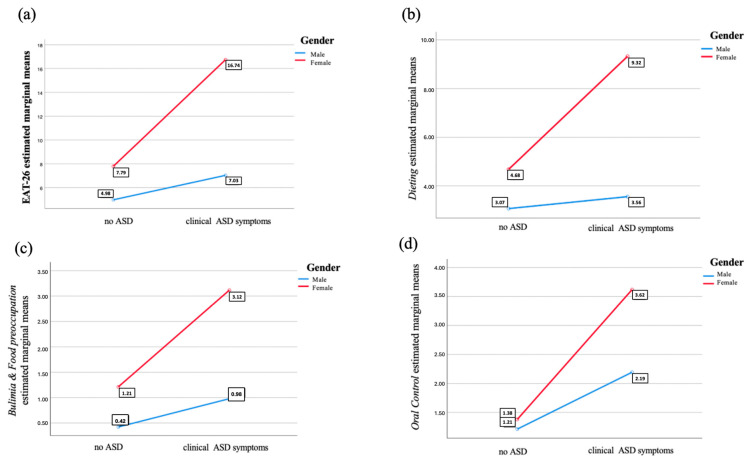
Effect of the presence of clinical ASD symptoms and gender on EAT-26 total (**a**) and do-main (**b**–**d**) scores.

As reported in Table 6a, results from the three two-way ANOVAs showed a significant effect of the presence of clinical ASD symptoms and of gender on EAT-26 (ANOVA n.2) and AICA-S (ANOVA n.1) total scores, while only an effect of ASD symptoms was reported for HQ-25 total scores (ANOVA n.3), in line with the results of the above reported *t*-test. Moreover, a significant interaction between gender and the presence of ASD was detected for AICA-S and EAT-26 total scores. In particular, while in both sexes the AICA-S total and EAT-26 total and domain scores significantly increase in subjects with above-threshold AdAS Spectrum scores, the increasing trend was higher among males for AICA-S and among females for EAT-26 (Figure 1 and Figure 2). A similar result was highlighted by the two two-way MANOVAs: the one performed with the HQ-25 domains as dependent variables showed a significant effect of gender (in line with the results of the above reported *t*-test) and ASD symptoms separately (gender: Wilks’ Lambda = 0.95, F = 19.09, *p* < 0.001; ASD symptoms: Wilk’s Lambda = 0.81, F = 92.80, *p* < 0.001) but not of their interaction (Wilks’ Lambda = 0.99, F = 0.46, *p* = 0.709), while a significant effect of both factors (gender: Wilks’ Lambda = 0.09, F = 37.33, *p* < 0.001; ASD symptoms: Wilk’s Lambda = 0.90, F = 40.20, *p* < 0.001) and of their interaction (Wilks’ Lambda = 0.97, F= 11.92, *p* < 0.001) was detected for EAT-26 domains (see Table 6b and Figure 2).

**Figure 3:**
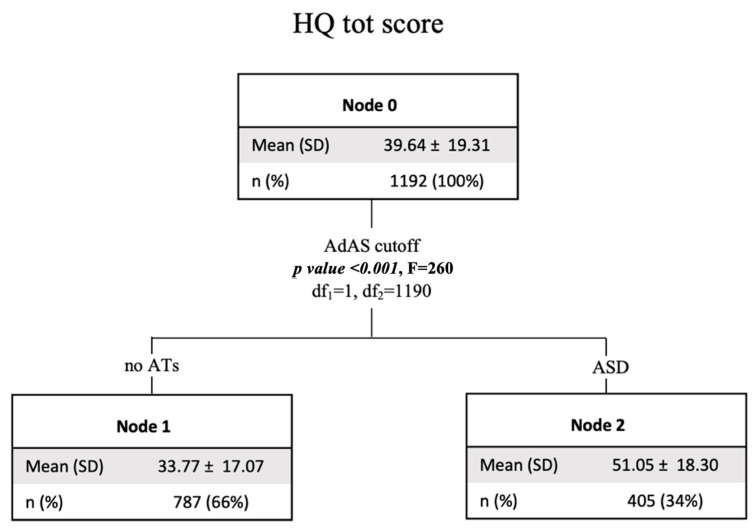
Tree decision model with HQ total score (risk for hikikomori) as dependent variable and gender and the presence of ASD clinical symptoms as independent variables. HQ: hikikomori questionnaire; AdAS Spectrum: adult autism subthreshold spectrum; ATs: autistic traits; ASD: autism spectrum disorder.

The decision tree models confirmed the MANOVA results: the one performed using the HQ-25 total score as a dependent variable and gender and the presence or absence of clinically relevant ASD symptoms as independent variables showed significantly higher HQ-25 scores in subjects with ASD clinical symptoms while no difference emerged based on gender (which in both the *t*-test and in the MANOVA, it influenced only the scores of the domains) (see Figure 3).

**Figure 4:**
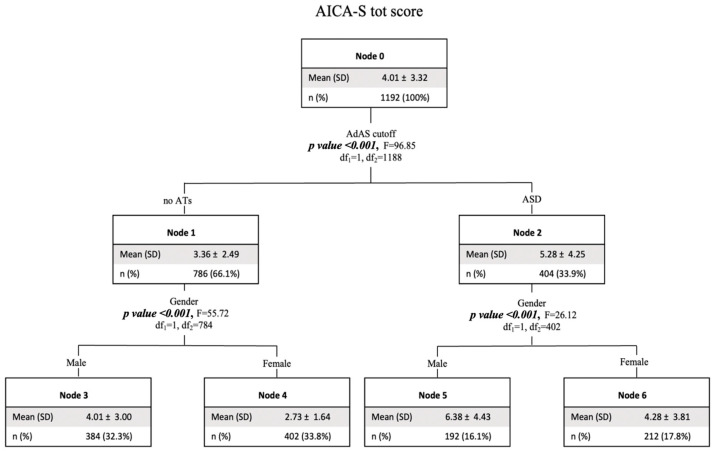
Tree decision model with AICA-S total score as dependent variable and gender and the presence of ASD clinical symptoms as independent variables. HQ: hikikomori questionnaire; AdAS: adult autism subthreshold spectrum; ATs: autistic traits; ASD: autism spectrum disorder.

A second decision tree model, that used the AICA-S total score as a dependent variable and gender and the presence or absence of clinically relevant ASD symptoms as independent variables, showed significantly higher AICA-S scores in subjects with ASD clinical symptoms, and subsequently, among both subjects with and without ASD symptoms, the possibility of having a higher AICA-S score was higher in males (see Figure 4).

**Figure 5:**
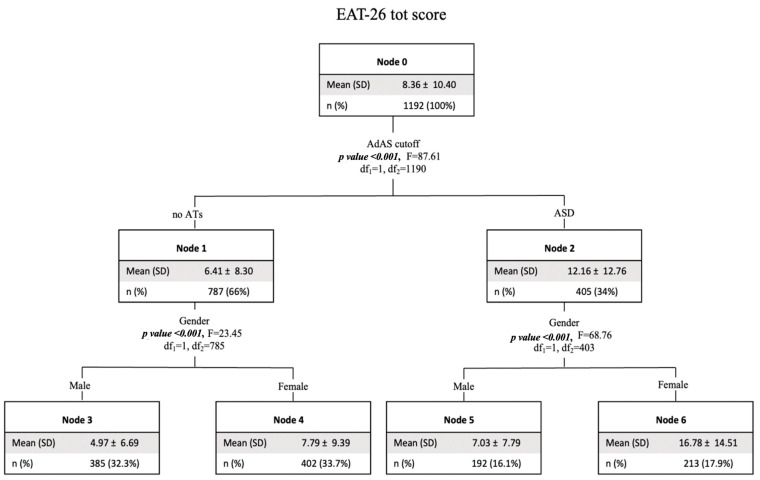
Tree decision model with EAT-26 total score as dependent variable and gender and the presence of ASD clinical symptoms as independent variables. EAT-26: eating attitude test; AdAS: adult autism spectrum; ATs: autistic traits; ASD: autism spectrum disorder.

Finally, one last decision tree model that used the EAT-26 total score as the dependent variable and gender and the presence of ASD symptoms as the independent variables revealed significantly higher EAT-26 scores in subjects with ASD clinical symptoms. Consequently, among both subjects with and without ASD symptoms, the possibility of having a higher EAT-26 score was higher in females (see Figure 5).

## 4. Discussion

In this study, we aimed to compare the prevalence of autistic traits, altered eating behaviors, videogame addiction, and pathological social withdrawal in university students enrolled in three-year bachelor’s degree courses, depending on socio-demographic variables such as gender, age, educational level of the parents, and the type of study course. Moreover, we aimed to evaluate the possible specific influence of gender and presence of autistic traits on social withdrawal, altered eating behaviors, and videogame addiction.

Our results highlighted some significant gender-based differences between domain and total scores of the psychometric instruments employed. In particular, males scored significantly higher than females in the AdAS Spectrum *empathy* domain, AICA-S total score, and HQ-25 *isolation* and *emotional support* domains. Interestingly, our results are in line with the available literature that has long since described gender-based differences in empathy. On the other hand, females scored significantly higher in AdAS Spectrum *hyper-hyporeactivity to sensory stimuli* domain, in all EAT-26 domains and total scores, and in the HQ-25 socialization domain.

Emotion recognition, emotional contagion, and emotion priming are just a few of the functional processes that constitute the phenomenon of empathy, which is commonly described as the ability to understand and share the internal states of others [66,67]. Over the years, many authors highlighted relatively stable gender differences in various measures of empathy [68,69,70,71,72]. Indeed, females have repeatedly been reported to be quicker and more precise not only in the identification of facial expressions [73,74] but also at recognizing bodily emotions [75,76]. Moreover, females were found to be more susceptible to emotional contagion than males and to exhibit more overt evidence of contagion for both positive and negative emotions [77]. Similarly, women are more likely than men to experience emotional contagion when helping others [78] and claim to regularly experience emotion contagion in their daily lives [79]. Lastly, regarding emotional priming, findings point to the possibility that whereas males are more sensitive to the joyful mood prime because they do not employ global processing by default, females appear to use it by default and are consequently less influenced by them [80,81]. Furthermore, empathy alterations have been historically considered a core feature of ASD and are due to a real difficulty in interpreting others’ emotions starting from facial expressions and body movements [82]. These emotional misunderstandings can lead the autistic subjects to engage in inappropriate reactions to the situation, caused by of a lack of emotional understanding of the others [82]. Despite this, it has long been demonstrated that even within subjects on the autistic spectrum (included high-functioning ones) there are significant gender-related differences and, in particular, autistic females showed higher empathy scores [6,83] and a greater ability to manage social relationships [84], which ultimately contribute to the difficulties of a diagnosis. On the other hand, sensory processing is the process through which humans are able to sense, interpret, and react to environmental sensory stimuli, and many authors have reported how up to 90% of subjects on the autism spectrum exhibit abnormal sensory profiles, which are characterized by atypical behaviors in response to sensory stimuli [85,86,87,88]. Notwithstanding the frequent occurrence of alterations in sensory processing in autistic subjects, to this date, the issue on how gender affects sensory processing has been scarcely addressed. Nevertheless, the available evidence seems to confirm our results, highlighting higher sensory processing alterations in females, reporting both higher scores on the “taste, smell, and touch response” scale [89] and a higher prevalence of lifetime sensory problems [90]. Interestingly, a significant gender-based difference in the overall prevalence of autistic traits did not emerge from our results. This appears somewhat in line with the current literature which has highlighted how the historical difference in prevalence in favor of the male gender could be due to the use of diagnostic methods based on a male-oriented conceptualization of the disorder as well as to the presence of a “female phenotype” of autism which presents itself with its own characteristics and conflicts with the conventional presentation, going often unrecognized [22,91]. In particular, the AdAS Spectrum questionnaire was also tailored to detect possible female-specific autistic traits. Furthermore, the reduced gender difference related to autistic traits in the overall sample may be consistent with recent data underlining how university students represent a population in which autistic features are particularly represented [13], and this may have further been emphasized by a recruitment bias: since the recruitment was carried out on a voluntary basis, subjects who identified with the characteristics described could be more motivated to complete the assessments.

The evidence of higher scores on AICA-S in the male population suggests a more marked tendency of the male sex towards the pathological use of online videogames and it aligns with the growing body of data on the matters that reported a greater tendency of the male gender in the development of videogame addiction, although not confirmed by all the studies [92,93,94,95,96]. Similarly, the evidence of higher scores on all EAT-26 domains and overall scores reported by the female portion of the sample confirms the commonly reported higher prevalence of feeding and eating disorders in the female population (5.5–17.9% vs. 0.6–2.4%) [97]. Finally, comparing HQ-25 scores, it appears that, while the total is similar between the two sexes, there are significant differences in some domains; in particular, the males reported higher scores in the *isolation* and *emotional support* domains, while females in the *socialization* domain. While hikikomori prevalence has been traditionally associated with a high prevalence among men, other studies and meta-analyses reported no significant difference between genders: our results align with the latter [98,99,100]. Moreover, some research described a more severe symptomatology with higher isolation and lower social support in males’ hikikomori, which could be in line with the higher scores of *isolation* and *emotional support* reported in our findings [98,99]. On the other hand, females often manifest greater difficulties and discomfort in socialization, as investigated by the HQ-25 *socialization* domain, such as feeling uncomfortable around people, disliking being seen by others, and having difficulties in joining groups [60,98]. Interestingly, those features also appear linked to the social phobic spectrum, which in turn has been reported to be more represented in adolescents and young adult females [99,100,101,102,103,104,105].

Results from the comparison of scores based on age highlighted that subjects over 23 years old scored significantly higher in the AdAS Spectrum *childhood and adolescence* domain, AICA-S total, and all HQ-25 domains and total. The choice of a threshold age of 23 years was motivated by fact that it is considered to separate subjects who are taking longer than expected to graduate and subjects who are up to date with their studies, for a bachelor’s degree. Although the significance of the scores difference in the *childhood and adolescence* domain was moderated, such results can be explained by some of the aspects that are investigated by the domain such as a difficulty in the educational environment present since early childhood and the dichotomy between difficulties and attitudes for different subjects [61]. Indeed, many studies reported greater university dropout rates in subjects with high autistic traits compared to the general population, due to challenges and opportunities within university environments, difficulty in university learning processes, difficulty interacting with friends, loneliness despite a desire for solitude, inaccessible extracurricular activities, and anxiety and sadness brought on by social expectations [106,107,108,109,110,111]. In addition, the risk of study dropouts or interruption has been previously linked to hikikomori tendencies. Difficulties in social and education environments, as well as struggles with finding employment due to economic stagnation, are risk factors to develop hikikomori behaviors [48,112,113,114]. A progressive social withdrawal would subsequently lead to a disruption of the academic path. On the other hand, social withdrawal may promote the use of online videogames, also as an alternative and low-demanding manner of maintaining social relationships [50,54,115]. Moreover, while the development of an internet gaming disorder may lead to a disengagement of other life activities, including education, both social withdrawal and pathological videogame use may cause greater levels of anxiety and depression and an overall reduction in the quality of social life [49]. These elements, combined with a difficulty in managing studies, interacting with peers, and in oral performance during exams, would seem to favor a greater risk of dropout during university studies or an overall delay in the graduation estimated time [116].

Interestingly, the results from the comparison of the scores based on the educational level of the parents highlighted higher scores in the *verbal* and *non-verbal communication* AdAS Spectrum domains as well as its total scores in subjects whose mothers had more than 13 years of schooling, while no difference emerged based on the educational level of the fathers. These data arise within the scientific literature that is currently controversial and requires further investigation. Indeed, while some studies excluded the relevance of parental education as a risk factor for the presence of higher autistic traits [117,118], others reported the presence of higher educational levels in parents of non-autistic children [119], and still others highlighted the presence of both a maternal and paternal higher educational degree, without gender difference, as a pre-natal risk factor for the development of ASD [120,121]. These data should be considered within the context of the presence of autistic-like features among first-degree relatives of ASD subjects, labeled in the literature as “Broad autism phenotype” (BAP) [6,122,123,124,125,126]. 

Intriguingly, scores obtained on the instruments also varied based on the type of university course attended. For instance, Sciences and Humanities students scored higher in AdAS Spectrum total score and most of its domains. Although specific investigation of autistic traits based on the type of academic field is still scant, these results overall align with data from previous studies that highlighted a higher prevalence of ASD and autistic features in Science course students and, to a lesser extent, in Humanities students [6,13,127]. This result is consistent with earlier research that highlighted a relationship between pursuing a Science degree and showing higher autistic features, which led to the hypothesis of a possible connection between autistic traits and scientific aptitude [6,128,129]. Moreover, as some authors suggested, students who pursue Humanities-oriented disciplines may express some autistic-like features such as a stronger interest in systems like languages and an analytic approach, choosing study pathways that are less work- or community-oriented and more theoretical [127,130]. Similarly, Science and Humanities students scored higher than Applied and Social Sciences students in the HQ-25, highlighting a greater risk of hikikomori in this populations. These results may be consistent with the previously reported link between hikikomori and the autism spectrum, which typically enhances difficulties in social interaction and the tendency towards social isolation [43,54,56,57,131,132,133]. Considering AICA-S, our results highlighted how Science students scored significantly higher than Applied and Social Sciences. To our knowledge, to this date, only a few studies have specifically evaluated the presence of videogame pathological use based on the type of academic field, not reporting any difference or highlighting a greater pathological gaming tendency among students of Art and Education with respect to Medical or Engineering ones [96,134]. However, considering that our findings highlighted a specific link between greater pathological videogaming and Science courses, it is also possible that Science students would be more frequently involved and driven to the use of computer and internet technologies, including for study purposes, while enjoying videogames more as a systematization model and as a creative use of technology. This increased interest may eventually enhance the risk of developing pathological use in vulnerable subjects. On the other hand, as in the case of hikikomori, these results may be in line with the link between autistic traits and the tendency towards videogame use reported in the scientific literature [59,135,136]. No significant differences emerged from the EAT-26 questionnaire, with the only exception of the *oral control* domain in which Humanities students scored higher than those of the other faculties. A previous study reported a similar result, hypothesizing that it could be explained by a higher interest in culture and attention to media in Humanities students, which may lead to a higher focus on beauty and body image [137].

Results from the two-way ANOVAs and MANOVAs highlighted how gender influences the score reported on AICA-S and EAT-26 but not the HQ-25 total scores, confirming our *t*-test results. On the other hand, the presence of autistic traits influenced the score obtained on all three scales. 

Moreover, there was also an effect of the interaction between presence of ASD symptoms and gender on both AICA-S and EAT-26. In particular, while, in both males and females, AICA-S and EAT-26 scores significantly rose with the increase of AdAS Spectrum scores, the AICA-S score increasing was higher in males, and the EAT-26 score rising was higher in females.

Findings from the classification tree analysis confirmed our data from the two-way ANOVAs, showing a discriminating effect of the presence of ASD symptoms, and not gender, on HQ-25. On the contrary, for both AICA-S and EAT-26, the presence of ASD symptoms was the first discriminating factor for higher scores, and subsequently gender, with higher scores reported in males for AICA-S and in females for EAT-26 in both subjects with and without ASD symptoms.

While in line with the current literature, which reported a role of autistic traits as a risk factor for the development of eating disorders and videogame addiction [59,135,136,138,139,140], our results also support the hypothesis of the existence of the gender-specific manifestation of the autism spectrum. Indeed, the recent literature contributed to the conceptualization of a female autism phenotype, characterized by a greater ability to camouflage social difficulties (eventually linked to an under-recognition of high-functioning ASD among females) and different kinds of specific interests, including a focus on food and diet [23,27,141,142,143,144,145,146]. In this framework, many authors hypothesized that eating behaviors, and particularly restrictive types such as anorexia nervosa, could represent a gender specific manifestation of ASD, proper of females, where restricted interests and repetitive behaviors are mainly linked to eating habits [27,140,147,148,149]. In this framework, a previous study from our group also reported a higher link between autistic traits and orthorexic tendencies among females than males [150]. Similarly, videogame addiction may be a more typical manifestation of ASD in males. While, as reported above, videogame addiction seems to be more frequent among males, the recent literature has highlighted a link between ASD and internet gaming disorder, hypothesizing that autistic features may promote the engagement of videogames, due to the low social demand of the activity and the tendency towards developing deep and restricted interests [54,59,135,136]. In addition, internet gaming may also be considered a safe space for communicating with others experiencing lower levels of distress, although enhancing the risk for developing a pathological use [54,59,135,136]. Contrarywise, hikikomori risk appeared to be discriminated exclusively by the presence of significant autistic traits, while gender did not exert any influence. This suggests a major role of autistic traits in determining the tendency of social withdrawal, in agreement with previous studies highlighting a link between autism spectrum and hikikomori [50,54]; in particular, autistic-like features such as deficits in social interaction, social communication, and unease in forming relationships may be a vulnerability factor for developing hikikomori behaviors [133]. One recent study from our group highlighted that, in this framework, difficulties in communication, empathy, and rumination are the autistic features mostly linked to hikikomori tendencies [55]. Autistic features can therefore act as a trigger for the development of hikikomori, as already suggested by the literature, in both sexes without major distinctions in the manifestation of social withdrawal [50,56,57].

Some limitations should be taken into account while evaluating these results. For instance, only university students enrolled in bachelor’s degree courses were included in the sample, limiting conclusions from being applied to a larger population. Second, participants were selected voluntarily, thus sample selection biases (e.g., an excess of subjects with higher levels of interest in the topic) should be considered. Thirdly, the use of self-reported questionnaires raises the possibility of symptom over- or underestimation. Fourthly, no specific inclusion or exclusion criteria were used in the sample recruitment procedure, which could partially influence the ability to complete the questionnaires. Finally, because the study was cross-sectional in nature, we are unable to make inferences regarding random or temporal relationships between the variables that were examined.

## 5. Conclusions

In conclusion, our results highlighted significant differences in the prevalence of autistic traits, social withdrawal tendencies, altered eating habits and pathological videogame use in university students based on gender, age, parents’ level of instruction, and the field of study. Moreover, our results support the role of autistic traits as a vulnerability factor for the development of a set of psychopathological conditions, in line with the rising hypothesis of a possible neurodevelopmental basis for different psychiatric disorders. Our results also seem to point out how gender could modulate this vulnerability, supporting the hypothesis of gender-specific phenotypes in the autism spectrum. While further stressing the link between the autism spectrum and extreme social withdrawal independently from gender, our findings support the hypothesis that conditions such as feeding and eating disorders and pathological videogame use may at times represent gender-specific manifestations of autism spectrum.

These results suggest the need to pay particular attention to mental health in university students, emphasizing how certain sub-populations (for example, subjects with high autistic traits) are more at risk of evolving into a psychopathological drift. It would therefore be suggested to use screening programs, exploiting cost-effective interventions such as self-administered questionnaires to highlight, monitor, and offer adequate support to those most at risk. In fact, an adequate early classification of such situations could have important implications on human capital which would make it economically advantageous from a social point of view to invest in the screening and improvement in the treatment of mental disorders of university students.

## Figures and Tables

**Table 1:** Comparison of AdAS Spectrum, AICA-S, EAT-26, and HQ-25 scores between males and females.

		Males Mean ± SD	Females Mean ± SD	*t*	*p*
**AdAS Spectrum**	**Child./Adolesc.**	8.70 ± 4.07	8.55 ± 3.98	0.65	0.518
	**Verb. comm.**	6.53 ± 3.35	6.34 ± 3.43	0.97	0.332
	**Non-verb. comm.**	11.32 ± 4.64	11.80 ± 4.95	−1.73	0.084
	**Empathy**	3.92 ± 2.59	3.39 ± 2.44	3.68	<0.001 *
	**Inflex. and routine**	15.40 ± 7.34	15.96 ± 7.60	−1.30	0.192
	**Restrict. Int. and rum.**	9.68 ± 4.56	9.64 ± 4.42	0.17	0.861
	**Hyper-hyporeact.**	4.86 ± 3.46	5.90 ± 3.77	−4.95	<0.001 *
	**Total Score**	60.43 ± 24.52	61.59 ± 24.95	−0.81	0.419
**AICA-S—Total Score**		4.80 ± 3.71	3.27 ± 2.70	8.10	<0.001 *
**EAT 26**	**Dieting**	3.23 ± 4.42	6.29 ± 7.43	−8.72	<0.001 *
	**Bulimia**	0.61 ± 1.70	1.89 ± 3.30	−8.50	<0.001 *
	**Oral control**	1.53 ± 2.62	2.15 ± 3.08	−3.70	<0.001 *
	**Total Score**	5.66 ± 7.13	10.91 ± 12.19	9.14	<0.001 *
**HQ 25**	**Socialization**	18.14 ± 9.77	19.71 ± 10.72	−2.65	0.008 *
	**Isolation**	13.55 ± 7.16	12.65 ± 7.15	2.19	0.029 *
	**Emotional support**	8.17 ± 5.00	7.07 ± 4.59	3.93	<0.001 *
	**Total Score**	39.86 ± 18.85	39.43 ± 19.75	3.83	0.702

*: statistically significant value (*p* < 0.05).

**Table 2:** Comparison of AdAS Spectrum, AICA-S, EAT-26, and HQ-25 scores between subjects of 23 years of age or older and subjects younger than 23.

		≥23 y.o. Mean ± SD	<23 y.o. Mean ± SD	*t*	*p*
**AdAS Spectrum**	**Child./Adolesc.**	8.93 ± 4.23	8.41 ± 3.85	2.15	0.032 *
	**Verb. comm.**	6.61 ± 3.55	6.31 ± 3.27	1.50	0.134
	**Non-verb. comm.**	11.63 ± 4.96	11.53 ± 4.70	0.35	0.728
	**Empathy**	3.73 ± 2.55	3.59 ± 2.51	0.98	0.328
	**Inflex. and routine**	15.76 ± 7.72	15.65 ± 7.30	0.25	0.820
	**Restrict. Int. and rum.**	9.74 ± 4.67	9.61 ± 4.36	0.49	0.622
	**Hyper-hyporeact.**	5.54 ± 3.86	5.30 ± 3.52	1.12	0.262
	**Total Score**	61.94 ± 25.97	60.39 ± 23.84	1.05	0.294
**AICA-S—Total Score**		4.40 ± 3.65	3.74 ± 3.04	3.30	0.001 *
**EAT 26**	**Dieting**	4.81 ± 6.23	4.81 ± 6.42	−0.00	0.998
	**Bulimia**	11.24 ± 2.64	1.29 ± 2.78	−0.30	0.767
	**Oral control**	1.97 ± 3.06	1.77 ± 2.75	1.20	0.230
	**Total Score**	8.49 ± 10.32	8.28 ± 10.46	0.34	0.736
**HQ 25**	**Socialization**	20.09 ± 10.85	18.15 ± 9.82	3.14	0.002 *
	**Isolation**	14.20 ± 7.49	12.31 ± 6.83	4.46	<0.001 *
	**Emotional support**	7.97 ± 4.88	7.35 ± 4.77	2.20	0.028 *
	**Total Score**	42.26 ± 20.36	37.81 ± 18.34	3.87	<0.001 *

*: statistically significant value (*p* < 0.05).

**Table 3:** Comparison of AdAS Spectrum, AICA-S, EAT-26, and HQ-25 scores based on the level of instruction of the father.

		13 Years of Schooling or Lower Mean ± SD	Schooling Higher than 13 Years Mean ± SD	*t*	*p*
**AdAS Spectrum**	**Child./Adolesc.**	8.52 ± 4.07	8.78 ± 3.95	−1.07	0.286
	**Verb. comm.**	6.38 ± 3.36	6.51 ± 3.44	−0.62	0.532
	**Non-verb. comm.**	11.51 ± 4.71	11.67 ± 4.96	−0.57	0.566
	**Empathy**	3.60 ± 2.48	3.70 ± 2.59	−0.65	0.517
	**Inflex. and routine**	15.46 ± 7.43	16.07 ± 7.54	−1.36	0.174
	**Restrict. Int. and rum.**	9.54 ± 4.52	9.86 ± 4.43	−1.19	0.233
	**Hyper-hyporeact.**	5.36 ± 3.63	5.47 ± 3.71	−0.51	0.609
	**Total Score**	60.38 ± 24.82	62.06 ± 24.64	−1.14	0.255
**AICA-S—Total Score**		4.05 ± 3.36	3.95 ± 3.26	0.49	0.622
**EAT 26**	**Dieting**	4.64 ± 6.16	5.08 ± 6.63	−1.15	0.249
	**Bulimia**	1.16 ± 2.62	1.43 ± 2.88	−1.62	0.106
	**Oral control**	1.86 ± 2.87	1.84 ± 2.90	0.10	0.920
	**Total Score**	8.15 ± 10.07	8.73 ± 10.91	−0.94	0.345
**HQ 25**	**Socialization**	18.84 ± 10.09	19.12 ± 10.63	−0.46	0.646
	**Isolation**	12.90 ± 6.07	13.37 ± 7.47	−1.08	0.279
	**Emotional support**	7.56 ± 4.79	7.67 ± 4.89	−0.37	0.712
	**Total Score**	39.30 ± 18.85	40.15 ± 20.04	−0.75	0.456

**Table 4:** Comparison of AdAS Spectrum, AICA-S, EAT-26, and HQ-25 scores based on the level of instruction of the mother.

		Middle School Diploma or Lower Mean ± SD	High School Diploma or Higher Mean ± SD	*t*	*p*
**AdAS Spectrum**	**Child./Adolesc.**	8.45 ± 4.08	8.82 ± 3.94	−1.60	0.111
	**Verb. comm.**	6.17 ± 3.36	6.73 ± 3.39	−2.84	0.005 *
	**Non-verb. comm.**	11.27 ± 4.79	11.92 ± 4.81	−2.34	0.019 *
	**Empathy**	3.52 ± 2.52	3.78 ± 2.52	−1.79	0.073
	**Inflex. and routine**	15.33 ± 7.56	16.11 ± 7.37	−1.78	0.075
	**Restrict. Int. and rum.**	9.47 ± 4.47	9.89 ± 4.49	−1.64	0.101
	**Hyper-hyporeact.**	5.30 ± 3.66	5.52 ± 3.66	−1.03	0.305
	**Total Score**	59.51 ± 25.07	62.78 ± 24.27	−2.27	0.023 *
**AICA-S—Total Score**		3.96 ± 3.30	4.07 ± 3.34	−0.59	0.552
**EAT 26**	**Dieting**	4.85 ± 6.51	4.77 ± 6.15	0.23	0.819
	**Bulimia**	1.23 ± 2.74	1.31 ± 2.70	−0.51	0.611
	**Oral control**	1.80 ± 2.80	1.91 ± 2.98	−0.63	0.529
	**Total Score**	8.31 ± 10.48	8.44 ± 10.32	−0.21	0.835
**HQ 25**	**Socialization**	18.75 ± 9.97	19.17 ± 10.67	−0.71	0.477
	**Isolation**	13.03 ± 6.93	13.13 ± 7.43	−0.25	0.840
	**Emotional support**	7.51 ± 4.78	7.70 ± 4.88	−0.69	0.492
	**Total Score**	39.29 ± 18.46	40.01 ± 20.26	−0.64	0.522

*: statistically significant value (*p* < 0.05).

**Table 5:** Comparison of AdAS Spectrum, AICA-S, EAT-26, and HQ-25 scores based on type of study course.

		Science Mean ± SD	Humanities Mean ± SD	Applied and Social Sciences Mean ± SD	F	*p*
**AdAS Spectrum**	**Child./Adolesc.**	8.98 ± 4.06	9.32 ± 4.06	8.12 ± 3.92	9.85	<0.001 °
	**Verb. comm.**	6.94 ± 3.47	6.64 ± 3.39	6.03 ± 3.29	8.60	<0.001 *
	**Non-verb. comm.**	11.92 ± 4.64	12.55 ± 4.90	10.94 ± 4.79	11.24	<0.001 °
	**Empathy**	3.99 ± 2.64	3.90 ± 2.57	3.33 ± 2.40	9.20	<0.001 °
	**Inflex. and routine**	16.19 ± 7.73	17.42 ± 7.35	14.66 ± 7.21	13.11	<0.001 °
	**Restrict. Int. and rum.**	10.01 ± 4.60	10.54 ± 4.24	9.08 ± 4.44	10.87	<0.001 °
	**Hyper-hyporeact.**	5.45 ± 3.71	6.39 ± 3.78	4.96 ± 3.50	13.42	<0.001 ^§^
	**Total Score**	63.48 ± 25.55	66.76 ± 24.32	57.13 ± 23.77	16.00	<0.001 °
**AICA-S—Total Score**		4.43 ± 3.75	3.85 ± 3.02	3.82 ± 3.13	4.24	0.015 *
**EAT 26**	**Dieting**	4.61 ± 6.24	5.13 ± 6.80	4.80 ± 6.21	0.50	0.609
	**Bulimia**	1.22 ± 2.73	1.47 ± 2.79	1.21 ± 2.69	0.85	0.429
	**Oral control**	1.81 ± 2.75	2.28 ± 3.37	1.70 ± 2.72	3.56	0.029 ^#^
	**Total Score**	7.99 ± 10.31	9.32 ± 10.98	8.20 ± 10.19	1.33	0.266
**HQ 25**	**Socialization**	20.06 ± 10.37	20.95 ± 10.69	17.43 ± 9.86	13.40	<0.001 °
	**Isolation**	14.23 ± 7.39	14.15 ± 7.25	11.94 ± 6.81	15.19	<0.001 °
	**Emotional support**	78.03 ± 5.09	7.52 ± 4.55	7.37 ± 4.76	2.13	0.119
	**Total Score**	42.33 ± 19.82	42.62 ± 19.65	36.74 ± 18.43	13.29	<0.001 °

* Science > Applied and Social Sciences; ^#^ Humanities > Applied and Social Sciences; ° Science and Humanities > Applied and Social Sciences; ^§^ Humanities > Science and Applied and Social Sciences; statistically significant value (*p* < 0.05).

**Table 6:** (**a**) Results from the three two-way ANOVAs with the AICA-S (ANOVA n.1), EAT-26 (ANOVA n.2), and HQ-25 (ANOVA n.3) total scores as dependent variables, and the sex and presence of ASD symptoms as independent variables. (**b**) Results from the two MANOVAs with the EAT-26 domains (MANOVA n.1) and HQ-25 domains (MANOVA n.2) as dependent variables and ASD symptoms and gender.

(**a**)						
**Source**	**Dependent Variable**	**Type III Sum of Squares**	**df**	**Mean Square**	**F**	***p***
**Corrected model**	AICA-S tot score	1753.06	3	548.35	61.05	<0.001 *
	EAT-26 tot score	19,994.05	3	6664.68	72.79	<0.001 *
	HQ-25 tot score	80,207.73	3	26,735.91	87.25	<0.001*
**Intercept**	AICA-S tot score	20,193.08	1	20,193.08	2109.76	<0.001 *
	EAT-26 tot score	89,242.67	1	89,242.67	974.81	<0.001 *
	HQ-25 tot score	1,920,126.04	1	1,920,126.04	6266.13	<0.001 *
**AdAS Spectrum**	AICA-S tot score	1024.612	1	1024.612	107.05	<0.001 *
	EAT-26 tot score	8144.00	1	8144.00	88.96	<0.001 *
	HQ-25 tot score	79,454.10	1	79,454.10	259.29	<0.001 *
**Gender**	AICA-S tot score	759.80	1	759.80	79.28	<0.001 *
	EAT-26 tot score	10,546.69	1	10,546.69	115.203	<0.001 *
	HQ-25 tot score	37.49	1	37.49	0.12	0.727
AdAS Spectrum*Gender	AICA-S tot score	44.45	1	44.45	4.64	0.31 *
	EAT-26 tot score	3198.09	1	3198.09	34.93	<0.001 *
	HQ-25 tot score	215.80	1	215.80	0.70	0.402
**Error**	AICA-S tot score	11,351.54	1186	9.57		
	EAT-26 tot score	108,759.93	1188	91.55		
	HQ-25 tot score	364,028.31	1188	306.43		
**Total**	AICA-S tot score	32,252.75	1190			
	EAT-26 tot score	212,144.00	1192			
	HQ-25 tot score	2,317,439.00	1192			
**Corrected total**	AICA-S tot score	13,104.60	1189			
	EAT-26 tot score	128,753.98	1191			
	HQ-25 tot score	444,246.04	1191			
(**b**)						
**Source**	**Dependent variable**	**Type III sum of squares**	**df**	**Mean square**	**F**	***p***
**Corrected model**	**EAT-26**					
	Dieting	5851.249	3	1950.416	55.099	<0.001 *
	Bulimia	1055.814	3	351.938	53.800	<0.001 *
	Oral control	923.864	3	307.955	40.764	<0.001 *
	**HQ-25**					
	Socialization	24,177.939	3	8059.313	93.782	<0.001 *
	Isolation	8701.218	3	2900.406	65653	<0.001 *
	Emotional support	1817.900	3	605.967	27.762	<0.001 *
**Intercept**	**EAT-26**					
	Dieting	28,414.208	1	28,414.208	802.701	<0.001 *
	Bulimia	2222.064	1	2222.064	339.685	<0.001 *
	Oral control	4684.829	1	4684.829	620.130	<0.001 *
	**HQ-25**					
	Socialization	445,139.154	1	445,139.154	5179.837	<0.001 *
	Isolation	209,237.079	1	209,237.079	4736.280	<0.001 *
	Emotional support	68,159.754	1	68,159.754	3122.690	<0.001 *
**AdAS Spectrum**	**EAT-26**					
	Dieting	1772.232	1	1772.232	50.066	<0.001 *
	Bulimia	419.191	1	419.191	64.081	<0.001 *
	Oral control	686.519	1	686.519	90.874	<0.001 *
	**HQ-25**					
	Socialization	23,274.886	1	23,274.886	270.837	<0.001 *
	Isolation	8407.777	1	8407.777	190.318	<0.001 *
	Emotional support	1415.341	1	1415.341	64.843	<0.001 *
**Gender**	**EAT-26**					
	Dieting	3651.092	1	3651.092	103.143	<0.001 *
	Bulimia	588.970	1	588.970	90.035	<0.001 *
	Oral control	166.252	1	166.252	22.007	<0.001 *
	**HQ-25**					
	Socialization	662.321	1	662.321	7.707	0.006 *
	Isolation	226.965	1	226.965	5.138	0.024 *
	Emotional support	282.005	1	282.005	12.920	<0.001 *
AdAS Spectrum*Gender	**EAT-26**					
	Dieting	1156.825	1	1156.825	32.680	<0.001 *
	Bulimia	128.870	1	128.870	19.700	<0.001 *
	Oral control	102.243	1	102.243	13.534	<0.001 *
	**HQ-25**					
	Socialization	41.766	1	41.766	0.486	0.486
	Isolation	9.081	1	9.081	0.222	0.638
	Emotional support	25.977	1	25.977	1.190	0.276
**Error**	**EAT-26**					
	Dieting	42,053.140	1188	35398		
	Bulimia	7771.351	1188	6.542		
	Oral control	8974.854	1188	7.555		
	**HQ-25**					
	Socialization	102,093.041	1188	85.937		
	Isolation	52,842.882	1188	44.178		
	Emotional support	25,930.781	1188	21.827		
**Total**	**EAT-26**					
	Dieting	75,468.000	1192			
	Bulimia	10,740.000	1192			
	Oral control	13,985.000	1192			
	**HQ-25**					
	Socialization	554,306.0000	1192			
	Isolation	265,319.000	1192			
	Emotional support	96,702.000	1192			
**Corrected total**	**EAT-26**					
	Dieting	47,904.389	1191			
	Bulimia	8827.164	1191			
	Oral control	9898.717	1191			
	**HQ-25**					
	Socialization	126,270.980	1191			
	Isolation	61,184.100	1191			
	Emotional support	27,748.681	1191			

*: statistically significant value (*p* < 0.05); df: degrees of freedom.

## Data Availability

All data generated or analyzed during this study are included in this published article.
